# Comparison of Coconut Water and Jordanian Propolis on Survival of Bench-dried Periodontal Ligament Cells: An *in vitro* Cell Culture Study

**DOI:** 10.5005/jp-journals-10005-1211

**Published:** 2013-10-14

**Authors:** Sanaa Najeh Al-Haj Ali, Suhad Al-Jundi, Nizar Mhaidat

**Affiliations:** Assistant Professor, Department of Orthodontics and Pediatric Dentistry, Qassim University, PO Box 6666, Qassim, Kingdom of Saudi Arabia, e-mail: dr.sanaa.alhajali@qudent.org; Professor, Department of Pediatric and Preventive Dentistry, Jordan University of Science and Technology, Irbid, Jordan; Associate Professor, Department of Clinical Pharmacy, Jordan University of Science and Technology, Irbid, Jordan

**Keywords:** Avulsion, Cell culture, Mature coconut water, Propolis, Dry storage

## Abstract

**Aim:** The aim of this study is to assess and compare the efficacy of Jordanian propolis and full concentration mature coconut water in their ability to preserve periodontal ligament (PDL) cell viability after exposure of PDL cells to up to 120 minutes dry storage.

**Materials and methods:** PDL cells were obtained from sound permanent first molars which were cultured in Dulbecco's Modified Eagles Medium (DMEM). Cultures were subjected to 0, 30, 45, 60, 90 and 120 minutes dry storage times then incubated with 100% mature coconut water, Jordanian propolis and DMEM for 45 minutes at room temperature (18-26°C). Untreated cells served as controls at each dry storage time tested. PDL cell viability was assessed by MTT assay. Statistical analysis of data was accomplished by using one-way analysis of variance complemented by Tukey test and the level of significance was 5% ( p < 0.05).

**Results:** Up to 60 minutes dry storage, no significant improvement on the percentage of viable cells was found from soaking in all tested media. On the other hand, soaking in mature coconut water only resulted in higher percentages of viable cells at >60 minutes dry storage. However, this improvement was not significant (p > 0.05).

**Conclusion:** Avulsed teeth which have been left dry for <45 minutes should be replanted immediately, whereas avulsed teeth which have been left dry for >45 minutes may benefit from soaking for 45 minutes in mature coconut water.

**How to cite this article:** Al-Haj Ali SN, Al-Jundi S, Mhaidat N. Comparison of Coconut Water and Jordanian Propolis on Survival of Bench-dried Periodontal Ligament Cells: An *in vitro* Cell Culture Study. Int J Clin Pediatr Dent 2013;6(3):161-165.

## INTRODUCTION

Avulsion is one of the most severe traumatic injuries characterized by complete displacement of the tooth outside the socket.^1^ Avulsion management focuses on replantation at the accident site.^2,3^ Unfortunately, this is rare to occur, as literature reported that replantation occurs most commonly between 1 and 4 hours after avulsion.^4^ Therefore, there are certain factors that should be considered prior to replantation including the extra-alveolar duration and the storage conditions.^5,6^ These factors are directly related to the favorable conditions which include preservation of PDL cells' viability and cementum integrity.^4^

The storage conditions include whether the avulsed tooth is stored dry or in a liquid storage medium. Dry storage causes death of PDL cells.^7^ Moreover, many literature reports correlated dry storage with an increased incidence of ankylosis and replacement resorption.^8-10^ Recently, coconut water and propolis were suggested as storage media for avulsed teeth.^2,3,11-13^

Propolis is a resinous hive substance produced by honey bees from collected plant products.^5^ The precise chemical composition of propolis is variable according to the plant source, the climate, season, year and location.^14^ Propolis gave more viable PDL cells than HBSS and milk after up to 24 hours of storage.^11^

The coconut (Cocus nucifera L) is a plant that generally flowers monthly.^15^ The ripe coconut is a large drupe that consists of a solid white kernel that is surrounded by a hard shell. Coconut water is found inside the cavity of the kernel. In young coconut, the fibrous coat is white and firm and the kernel is thin and jelly-like.^16^ Coconut is usually harvested at around 9 months of age, beyond this time the kernel hardens and the volume of water begins to decrease. In this case, the coconut becomes more mature.^15^ Recent reports showed that coconut water kept significantly more PDL cells viable, compared to HBSS or milk.^3,12^ Coconut water even had significantly more PDL cells viable compared with propolis after 30 minutes of dry storage.^3^ A recent study using cell culture model demonstrated that full concentration mature coconut water was superior to 50% dilutions obtained from either young or mature coconuts.^17^

Up to date, no comparison has been made over the efficacy of propolis and coconut water at various dry storage times and at room temperature using an *in vitro* cell culture model. Moreover, Jordanian propolis has never been investigated to see whether it has promising results or not. Therefore, the main aim of this study is to assess and compare the efficacy of Jordanian propolis (in different solvents) with full concentration mature coconut water in their ability to preserve PDL cell viability after exposure of PDL cells to up to 120 minutes dry storage using an *in vitro* culture model.

## MATERIALS AND METHODS

The *in vitro* cell culture model involving PDL cells was approved by the institutional review board (IRB) and the ethical committee of the faculty of medicine at Jordan University of Science and Technology.

### Preparation of Propolis Solutions

Hundred and forty grams of crude Jordanian propolis were collected in February, 2009 from Al-Salt area*.* The propolis samples were de-waxed by placing them in a water bath at 70°C for 1 hour. A pure ethanolic method for extraction of Jordanian propolis was used since ethanol was the most commonly used vehicle in dental literature.^2,3,14^ Briefly, de-waxed propolis was extracted five times with 400 ml aliquots of 70% ethanol (EtOH). The ethanolic extracts were vacuum filtered with cellulose acetate filter paper (0.45 μm) (VIVID Separation and Filtration, USA) and evaporated until a dark brown resinous product was achieved.

Two propolis solutions were prepared at 10% concentration using EtOH (70%) and PG as solvents, respectively. Both propolis solutions were prepared using a modified method to that described by Sonmez et al.^14^Briefly, 6 gm of the resinous product were weighed and dissolved in 60 ml EtOH (70%) or 60 ml PG in a Sonicator for 3 hours. Then, both propolis solutions were filtered.

Serial dilutions of the prepared propolis solutions were made in growth medium (DMEM; Bio Whittaker, Belgium) and piloted until nontoxic final dilutions were reached (0.02% or 1/512 dilution).

### Primary Culture of Human Periodontal Ligament Cells

The PDL tissue explants were obtained from two fully erupted sound maxillary first permanent molars extracted from an 11-year-old patient for orthodontic reasons. Written consent was obtained before extraction of potential teeth. The teeth were extracted as atraumatically as possible and they were immediately transferred to a 50 ml centrifuge tube containing 10 ml of DMEM, 10 ml of fetal bovine serum (FBS) and an antibiotic solution consisting of 300 μl of penicillin-streptomycin mixture and 300 μl of amphotericin B ( all obtained from Bio Whittaker, Belgium). The extracted teeth were immediately transported to the tissue culture laboratory where the middle third of the root surface was mechanically scraped with a number 15 scalpel using aseptic techniques to obtain samples of PDL tissue. The PDL tissue was diced into small tissue explants of 1 mm^3^. The tissue explants were placed into tissue culture flasks (25 cm^2^) and they were incubated with DMEM containing glucose (4.5 gm/l), penicillin (100 μg/ml), streptomycin (100 μg/ml), amphotericin B (0.25 μg/ml) and 10% heat inactivated FBS.

Cells were grown at 37°C in a humidified atmosphere of 5% CO_2_ in air. After 4 and 5 weeks, cells reached confluence then they were detached after trypsinization (0.25% trypsin with ethylene diaminetetra-acetic acid (EDTA) obtained from Sigma-Aldrich, USA) for 5 to 10 minutes and transferred to larger flasks (75 cm^2^) for continued growth.

Subconfluent cultures were characterized to assure their PDL cell phenotype by the presence of alkaline phosphatase. Culture medium was renewed every 2 to 3 days until cells reached 80 to 90% confluency.

Cells used for the experiments proliferated in logarithmic phase between the 7th and 15th passages. Cells were seeded at a density of 1.0×10^4^ cells/well in 100 μl full growth medium in 96-well plates. The plates were incubated at 37°C in a humidified atmosphere of 5% CO_2_ in air for 48 hours.

### Exposure of PDL Cultures to Tested Media

On the day of treatment, the culture medium was drained from each well and the cells were rinsed with phosphate buffered saline solution (PBS). Cultured cells were distributed into six experimental groups corresponding to the dry storage time tested.

In each experimental group, PDL cells were bench-dried to a certain period (0, 30, 45, 60, 90 or 120 minutes) in a separate 96-well plate, then they were incubated with 200 μl of 100% mature coconut water obtained from an Indian coconut, 0.02 % dilution of Jordanian propolis dissolved in EtOH (70%), 0.02% dilution of Jordanian propolis dissolved in PG and DMEM for 45 minutes at room temperature. Untreated cells served as controls in each dry storage time tested. In addition, cells treated with the same dilution of the solvent (EtOH (70%) or PG) in DMEM used for propolis solutions were included in the experiment. Experiments were carried out in quadruplicate wells and they were repeated on two different occasions.

### Assessing the Viability of Cells by MTT Assay

A 5 mg/ml solution of MTT ( Sigma Aldrich, USA) in PBS was produced by dissolving 500 mg of MTT in 100 ml of PBS, then the solution was filtered with 0.45 μm cellulose acetate filter paper) ( VIVID separation and filtration, USA) to ensure sterilization.

After incubation with experimental media, all media were removed and the PDL cells were washed twice with PBS, then 100 μl of DMEM and 10 μl of MTT solution were added to all wells and all plates were incubated for 4 hours at 37°C. The supernatant was then eliminated and 100 μl of DMSO solvent (Sigma Aldrich, USA) was added to each well*.* The plates were then incubated for 20 minutes at 37°C.

The absorbance at 570 nm was measured with a microtiter plate reader (Tecan, Austria) with absorbance at 650 nm used as a reference*.* The mean absorbance value of untreated wells at 0 minutes dry storage was used to indicate the 100% viability value. The percentage of viable cells was calculated as the relative absorbance of tested wells *vs* untreated wells at 0 minutes dry storage as follows:

Percent (%) cell viability = mean absorbance of tested wells/mean absorbance of untreated wells at 0 minutes dry storage) *100.

### Statistical Analysis

Statistical analysis of data was accomplished by using oneway analysis of variance complemented by Tukey test. The level of significance was 5% (p < 0.05).

## RESULTS

The mean absorbance and percentage of viable cells are shown in Table 1. There was a significant drop in the percentage of viable cells for untreated control cells, mature coconut water and PG propolis solution as the dry storage time progressed from 0 to 90 minutes ( p < 0.05), whereas the drop was insignificant from 90 to 120 minutes. On the other hand, the drop in the percentage of viable cells was only significant from 0 to 60 minutes dry storage times for EtOH propolis solution and DMEM.

The efficacy of mature coconut water was found to be significantly better than all tested media at all dry storage times tested except DMEM at 0 minutes and PG propolis solution at 120 minutes. In the latter circumstances, the differences were insignificant.

When mature coconut water and untreated control cells are compared, no difference in the percentage of viable cells was observed at 0 minutes. At 30 minutes, mature coconut water was significantly worse than untreated control cells, whereas at 45 minutes, the difference was insignificant. On the other hand, mature coconut water was better than untreated control cells at 60 to 120 minutes dry storage times; however, the differences were insignificant.

**Fig. 1:**
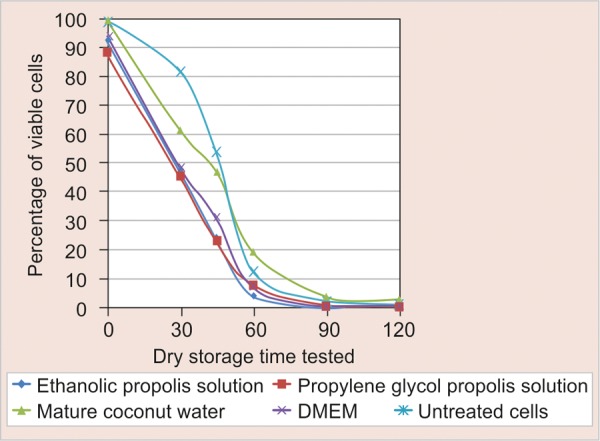
Percentage of viable cells according to the dry storage time tested

No significant difference was found between both propolis solutions at all dry storage times tested. Moreover, both propolis solutions were significantly worse than untreated control cells at 30, 45 and 90 minute dry storage times. EtOH propolis solution was also significantly worse than untreated control cells at 120 minutes. However, the remaining differences were insignificant. Figure 1 shows the percentage of viable cells according to the dry storage time tested.

**Table 1:** **Table 1:** Mean absorbance and percentage of viable cells in tested media and untreated control cells at various dry storage times tested

*Medium*	*Dry storage time*										
	0		30		45		60		90		120
	Percentage of viable cells ( mean absorbance)										
EtOH propolis solution	93.04		47.68		23.87		3.85		0		0
	(0.7555)		(0.4018)		(0.2161)		(0.06)				
PG propolis solution	88.22		45.19		22.91		7.36		0.41		0.23
	(0.7184)		(0.3829)		(0.2091)		(0.0879)		(0.0336)		(0.0323)
Mature coconut water	100		61.32		46.93		19.06		3.45		2.58
	(0.8143)		(0.5021)		(0.390)		(0.1726)		(0.0509)		(0.0441)
DMEM	95.05		48.43		30.85		6.6		0		0
	(0.7756)		(0.4121)		(0.2749)		(0.0859)				
Untreated cells	100		81.65		53.55		12.12		2.23		1.06
	(0.8143)		(0.6711)		(0.452)		(0.129)		(0.0519)		(0.0428)

## DISCUSSION

The presence of a dry storage time following avulsion and its effect on the prognosis following replantation is a field that has been studied thoroughly. However, most reports in the literature focused on two major dry storage times which are 30 and 60 minutes. Sixty minutes dry storage time has long been thought as a critical time point at which no viable PDL remains and root surface treatment of the avulsed tooth should be initiated before replantation. The results of this study showed that the percentage of viable cells dropped continuously with increasing dry storage time; however, this drop was not in an exact linear fashion. Moreover, most of the cells lost their viability in the period 45 to 60 minutes; cell viability dropped from 53.55% at 45 minutes to 12.19% at 60 minutes, therefore, at 60 minutes dry storage time, a significant drop off in the number of viable cells was demonstrated. At 90 and 120 minutes, the percentage of viable cells was almost negligible, which is in agreement with the findings of Doyle et al^18^ and Soder et al.^19^

In the dental literature, various studies used enzymatic disaggregation procedures that involved trypsinization,^19,20^treatment with dispase and collagenase,^2,3,12^ or collagenase and protease^13^ in order to collect PDL cells. In addition, several studies used manual staining methods such as Trypan blue exclusion,^2,3,11,12,18^ neutral red^19^ and fluorescein diacetate to quantify viable cells.^20^ Enzymatic disaggregation procedures may damage some cells or lead to incomplete disaggregation.^21^ Whereas, manual staining methods of viable cells may not give any idea about the actual physiologic health or metabolic capabilities of the cell which are likely critical for the prevention of resorption sequelae postreplantation.^2^

In this study, the number of viable cells was calculated by means of a highly calibrated and reliable microtiter plate reader. In addition, MTT assay which is a widely used assay to measure viability with the advantages of being sensitive (detecting as few as 10^3^ viable cells/ml) and accurate, with color development strongly correlating with cell numbers^22^was used.

Recently propolis and coconut water were suggested as storage media. Brazilian propolis has widely been investigated and it has proven to have certain components that play an important role against inflammation (artepillin-C at a concentration of 5-11%). Recently, one report suggested that Brazilian propolis inhibits osteoclastogenesis and osteoblast activation in tissue culture.^23^

The results of this study showed that soaking in propolis (both solutions) gave inferior results than untreated cells at all dry storage times tested. At 60 to 120 minutes, ethanolic propolis solution was one of the worst media; by 90 minutes, all PDL cells were nonviable. Therefore, soaking in propolis for 45 minutes at room temperature caused negative effects on the viability of PDL cells. It can be hypothesized that soaking in propolis should be extended more than 45 minutes to be beneficial. This could be true when the findings of Mori et al^24^ and Ozan et al^11^ are considered.

Mori et al found that soaking in propolis for 6 hours prior to replantation gave better healing results following replantation than soaking for 60 minutes.^24^ On the other hand Ozan et al found that soaking in propolis gave significantly superior results than HBSS and milk when the soaking period was **>**3 hours.^11^ Moreover, it can be hypothesized that the negative effects of propolis can be reversed when the soaking period is extended. On the other hand, the results of this study showed that soaking in coconut water at <45 minutes dry times was not beneficial, since untreated cells were better in this period (except at 0 minute dry storage time), this finding is in part in agreement with Gopikrishana et al who found that after 30 minutes dry time, coconut water was significantly worse than the positive control where the teeth were assessed immediately without soaking. In this study, coconut water was significantly better than propolis at 30 minutes dry storage time. The same finding was reported by Gopikrishana.^3^ Untreated control cells exposed to 30 minutes of dry storage time were not included for comparison in the study by Gopikrishana.^3^However, they were included in this study.

It can be hypothesized from the results of this study that after 45 minutes dry storage, soaking in mature coconut water could maintain the viability of the healthy PDL cells. However, the ability to revitalize damaged cells or stimulate the remaining cells was unlikely to be achieved under the conditions of this study. This hypothesis could be true if the osmolarity of coconut water was considered (372 mOsm/l). Therefore, it is most likely that coconut water had the ability to maintain the integrity of the cell membrane.

## SUMMARY

Within the parameters of this study, it appears that for the highest success rates, avulsed teeth that have been left dry for <45 minutes should be replanted immediately without soaking, whereas avulsed teeth which are left dry for >45 minutes may benefit from soaking for 45 minutes in mature coconut water. However, further research is needed to confirm this finding, since actual improvement in the prognosis of an avulsed tooth cannot be determined without clinically simulated avulsion studies on animal models to validate the results of *in vitro* studies. Further research is also needed to study and compare propolis obtained from different regions and from wet and dry seasons in Jordan.
